# Systematic review of efficacy with extending contraceptive implant duration

**DOI:** 10.1002/ijgo.12696

**Published:** 2018-11-22

**Authors:** Lauren Thaxton, Antonella Lavelanet

**Affiliations:** ^1^ Department of Obstetrics and Gynecology University of New Mexico School of Medicine Albuquerque NM USA; ^2^ Department of Reproductive Health and Research and UNDP‐UNFPA‐UNICEF‐WHO‐World Bank Special Programme of Research Development and Research Training in Human Reproduction (HRP) World Health Organization Geneva Switzerland

**Keywords:** Contraception, Duration, Etonogestrel, Implanon, Implant, Jadelle, Levonorgestrel

## Abstract

**Background:**

Extending contraceptive implant duration of use increases accessibility by maximizing the lifetime of devices.

**Objectives:**

To review the contraceptive efficacy during extended use of progestin implants.

**Search strategy:**

PubMed and EMBASE were searched for articles in any language, 1996–2017, utilizing terms for devices and contraceptive efficacy.

**Selection criteria:**

Randomized clinical trials (RCTs), cohort studies, and case–control studies were included; abstracts, posters, and presentations were excluded. Studies evaluating Norplant and implants currently in pre‐marketing trials were excluded.

**Data collection and analysis:**

Titles and abstracts of articles were reviewed; those that met inclusion and exclusion criteria underwent full text review and data abstraction.

**Main results:**

The search identified 2951 articles; six met inclusion and exclusion criteria. Five studies evaluated the etonogestrel implant (Implanon), and one the levonorgestrel implant (Jadelle). One RCT randomized to method, not duration; the remaining studies were prospective cohort studies. Three studies analyzed efficacy among women beyond currently approved duration separately. All studies were of poor to fair quality by United States Preventative Services Task Force (USPTF) grading. Limitations include lack of generalizability and control of important confounders.

**Conclusion:**

These studies provide limited data for extended duration of contraceptive implants.

## Abstract 2

This review provides evidence for contraceptive efficacy of progestin‐only implants beyond currently approved durations. There is insufficient evidence to uniformly recommend extension of these devices.

## 1 INTRODUCTION

Progestin‐only implants are highly effective contraceptive methods with less than 1% of women experiencing an unintended pregnancy in the first year of use.^1^, ^2^, ^3^ Women of all ages are interested in the contraceptive implant because of its long duration of action, reversibility, and efficacy^4^, ^5^, ^6^ (also C. Kennedy, unpublished data, 2017). Implants can also be highly cost‐effective.^7^, ^8^ Contraindications to implants are uncommon owing to the lack of an estrogen component.^9^ Implants exhibit high continuation rates and represent an increasing proportion of the contraceptive method mix.^10^, ^11^

Implants have three probable mechanisms of action: suppression of ovulation, thickening of cervical mucus, and endometrial atrophy.^3^ Two subdermal progestin implants currently available globally include etonogestrel (ENG)‐releasing devises, including Implanon and Nexplanon (Merck, Kenilworth, NJ, USA) and levonorgestrel (LNG)‐releasing devices, including the Sino‐Implant II (Dahua Pharmaceutical, Shanghai, China) and Jadelle (Bayer, Berlin, Germany).^12^, ^13^ The only difference between the two ENG implants is that Nexplanon contains a radiopaque dye used to locate the device in the event that it cannot be palpated. The key aspects of product information for each of these devices is included in Table 1.

**Table 1:** Description of devices

Device brand names	Manufacturer	Progestin	Rod	Recommended duration of use, y
Implanon	Merck	68 mg etonogestrel	1	3
Nexplanon^a^	Merck	68 mg etonogestrel	1	3
Sino‐Implant (II)	Shanghai Dahua Pharmaceuticals	75 mg levonorgestrel	2	4
Jadelle	Bayer	75 mg levonorgestrel	2	5

a Radio‐opaque device.

Pharmacokinetic studies provide information about the serum levels exhibited throughout use of contraceptive implants as well as what levels are required to inhibit ovulation. Prior work indicates that ovulation is suppressed at serum ENG levels greater than 90 pg/mL.^14^ McNicholas et al.^15^ found that median ENG levels at the end of 3 years of use of the implant are beyond this value at 207.7 pg/mL (range 63.8–802.6 pg/mL). Median serum ENG levels remain high at 166.1 pg/mL (range 25–470.5 pg/mL) at 4 years of use and 153.0 pg/mL (range 72.1–538.8 pg/mL) at 5 years of use.^15^ Furthermore, this study failed to show a difference in median serum ENG levels across body mass index (BMI) groups, including obese women, at the end of the fifth year of implant use.^15^ However, women may exhibit wide ranges of ENG, with some values below 90 pg/mL, as McNicholas found in 23 participants (written communication, July 2017).

Among women using the LNG implant, variations in serum LNG levels exist. Sivin et al.^16^ measured serum LNG concentrations among women extending duration. Pregnancies were identified in five women using the Jadelle LNG implant beyond 5 years. In all women, the serum LNG levels fell to or below 180 pg/mL, indicating that this may represent a threshold below which contraceptive efficacy cannot be guaranteed. Further analysis revealed that study site, body weight and ponderal index, and duration of use were the most important variables affecting serum LNG levels.^16^

Importantly, the pharmacokinetic data on ENG and LNG have revealed that serum concentrations between participants and even within the same participant over multiple collections vary widely.^15^, ^16^ Among women who have serum point values of progestin below presumed thresholds, pregnancies may not occur. It remains unclear what progestin level is needed to provide effective contraception, given the implant's other mechanisms of action including cervical mucus thickening and endometrial atrophy.

Barriers to obtaining a progestin‐only implant are reported among women who desire them, including access to trained professionals for placement and removal, and high up‐front device costs. Efforts have been made to take advantage of convenient opportunities for placement, such as immediately post pregnancy.^17^ Extending the duration beyond the currently approved length of use, if shown to be efficacious, could offer another means to enhance accessibility by maximizing the lifetime of the implant.

The purpose of this systematic review is to examine current evidence to answer the question: “What is the contraceptive effectiveness of progestin implants beyond currently approved durations of use (i.e. extended use)?”

## 2 MATERIALS AND METHODS

This review uses the Preferred Reporting Items for Systematic Review and Meta‐Analyses (PRISMA) guidelines for reporting.^18^

Our primary question is: among women using a contraceptive implant, are pregnancy rates different for women who continue using the same device beyond its currently approved duration from pregnancy rates among women who initiate use of a new contraceptive implant at its expiration date? Because we did not anticipate identifying many studies that met this primary question, we also included studies that assessed pregnancy rates during any duration of extended use of contraceptive implants.

We included studies published between January 1, 1996, and December 31, 2017, as US Food and Drug Administration (FDA) approval of the Jadelle implant occurred in 1996 and all other devices were developed and released after this year. The search was not restricted with regard to country or language. Studies evaluating levonorgestrel implants (Jadelle, currently approved for 5 years of use and Sino Implant, currently approved for 4 years of use) and etonogestrel implants (Implanon or Nexplanon, currently approved for 3 years of use) were included. We included randomized clinical trials (RCTs), cohort studies (comparative and noncomparative), case–control studies, and pharmacokinetic trials with some measure of contraceptive efficacy of one of the four devices specified beyond the currently approved duration.

We excluded from this review studies evaluating the levonorgestrel implant Norplant, as this device is no longer available, as well as contraceptive implants currently in pre‐marketing trials. Conference abstracts, posters, and oral presentations were excluded.

We searched published studies among PubMed and EMBASE. To locate articles, we used the following search terms for devices, developed together with a Health Science Librarian: “levonorgestrel”, “norplant”, “Jadelle”, “etonogestrel”, “Implanon”, “Nexplanon” and “sino implant,” as well as search terms for pregnancy outcomes (to assess contraceptive failure) and study designs including clinical trials or cohort studies (full search strategy available recorded in File S1).

The title and abstract from each article was independently evaluated by two reviewers (LT and AL) to determine if the article met inclusion/exclusion criteria. The full text of the article was obtained if reviewers judged a citation as potentially eligible; the full text was screened for eligibility by both reviewers. No discrepancies were noted. Reasons for exclusion, including number of duplicates, were documented (File S2).

Reviewers abstracted the following information from the remaining articles: study title, authors, publication year, funding source, study design, population size, duration of extended use, type of implant, and number of contraceptive failures. The findings from each study were summarized and synthesized narratively; we did not calculate summary measures of association.

Using the United States Preventative Services Task Force (USPSTF) guidelines, each article's study quality was rated as “poor,” “fair,” or “good”.^19^ This rating was based on methodological quality as assessed by the reviewers along the following domains: assembly of appropriate comparison groups (including adequate description and execution of randomization in RCTs and assessment of potential confounders in cohort studies such as participant age); maintenance of comparable groups and participant attrition during the study; and description of how pregnancy was assessed. Additionally, quality of data analysis and interpretation was assessed according to whether adjustments were performed for confounders, and whether contraceptive efficacy was measured cumulatively or analyzed separately among women beyond approved duration.

## 3 RESULTS

**Figure 1:**
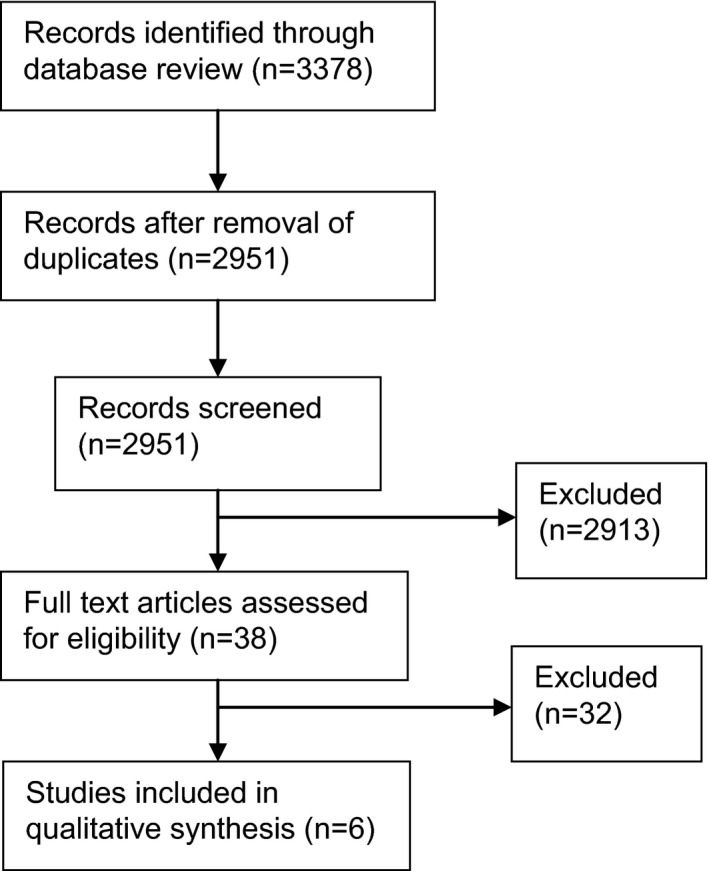
Flow diagram of study selection.

We identified 2951 citations and selected 38 articles for full‐text screening (Fig. 1). Thirty‐two studies were excluded because they did not meet inclusion or exclusion criteria (File S2). We included six published studies in this systematic review, resulting in a total of 1075 women who self‐selected extended duration of their contraceptive implant in a number of countries, including Brazil, Chile, Dominican Republic, Hungary, Thailand, Turkey, Zimbabwe, USA, and China^15^, ^16^, ^20^, ^21^, ^22^, ^23^(Table 2).

**Table 2:** Evidence

Author, year	Study design	Location	Extended method continuation	Loss to follow‐up	Implant type	Duration	Pregnancy outcomes and assessment	Strengths	Weaknesses	Quality
McNicholas et al. 2017^15^	Noncomparative cohort	USA	n=223 completing 3rd year	5% at 24 mo	1 rod ENG	Up to 2 y extended use	Year 4: Zero pregnancies per 100 woman years	Assessment of ancillary methods of contraception (barrier methods etc.) and comorbidities that may affect fertility (BMI, age)	Small sample size completing each additional year	II‐3, Fair
			n=102 completing 4th year				Year 5: Zero pregnancies per 100 woman years	Detailed description of study population provided	Self‐selected cohort extending duration	
							Assessment: Self‐report (with validation through medical record review where possible)	Study results generalizable with diverse demographics of study population	Pregnancy assessment – validation only where possible	
Ali et al. 2016^20^	Noncomparative cohort analysis from RCT	Brazil, Chile, Dominican Republic, Hungary, Thailand, Turkey, Zimbabwe	n=311 completing 4th year	2%	1 rod ENG	Up to 2 y extended use	Year 4: Zero pregnancies per 100 woman years	Detailed description of study population provided	Small sample size completing each additional year	II‐3, Fair
			n=204 completing 5th year				Year 5: Zero pregnancies per 100 woman years	Study results generalizable with multiple study sites	High implant discontinuation rate over extended duration (48%)	
							Assessment: Urine pregnancy testing if suspicion of pregnancy	Pregnancy assessment well defined		
								Low loss to follow‐up		
Yao and Du, 2003^21^	Nonrandomized, comparative, prospective observational	Not discussed	n=51 completing the 4th year	0%	1 rod ENG	Up to 1 y extended use	Zero pregnancies	Unable to assess generalizability	Small sample size completing each additional year	II‐3, Poor
							Assessment: No information		No assessment for potential confounders	
									Self‐selected cohort extending duration	
									No comparison group	
Sivin et al. 2001^16^	Multicenter, pharmacokinetic trial	USA	n=43 completing 6th year	No information	2 rod LNG	Up to 2 y extended use	2 pregnancies in the 5th year and 3 in the 7th year	Multiple study sites	Small sample size completing each additional year	II‐3, Poor
		non‐USA	n=39 completing 7th year				Assessment: No information		Self‐selected cohort extending duration	
									No comparison group	
Zheng et al. 1999^22^	Noncomparative, multicenter prospective observational	China	n=151 completing 4th year	1%	1 rod ENG	Up to 1 y extended use	Zero pregnancies	Assessment of appropriate confounders including: age (age at enrollment was <35 y old)	Small sample size completing each additional year	II‐3, Poor
							Assessment: Urine pregnancy testing if suspicion of pregnancy	Pregnancy assessment well defined	Self‐selected cohort extending duration	
									No comparison group	
									Lack of generalizability secondary to limited demographic data and all study sites within one country	
Kiriwat et al. 1998^23^	Noncomparative cohort	Thailand	n=47 completing 4th year	8%	1 rod ENG	Up to 1 y extended use	Zero pregnancies	Pregnancy assessment well defined	Small sample size completing each additional year	II‐3, Poor
							Assessment: Urine pregnancy testing if suspicion of pregnancy		No assessment for potential confounders	
									Self‐selected cohort extending duration	
									No comparison group	
									Lack of generalizability secondary to limited demographic data	

Abbreviations: ENG, Etonogestrel; LNG, Levonorgestrel.

We did not identify any studies that met the inclusion criteria for our primary question, comparing women who continued use of an implant after the approved duration with women who initiated a new implant after completing use through the approved duration. All six studies were noncomparative cohort analyses assessing pregnancy rates during extended duration of implant use. Five studies evaluated the ENG implant (Implanon), and one evaluated the LNG implant, Jadelle. No published studies evaluated the Sino‐Implant II. No studies separately evaluated Nexplanon; however, this device has the same dose of ENG and pharmacokinetic properties as Implanon and is assumed to behave similarly. Two studies were sponsored by the manufacturer of the single‐rod ENG implant. One study was published in Chinese and a translator was used to extract data.^21^ Three studies separately evaluated contraceptive failure over the time frame of extended duration,^15^, ^16^, ^20^ while the other three studies reported on cumulative pregnancy rates over the total time period of implant use (i.e. approved duration plus extended duration).

One publication represents interim analysis of an ongoing clinical trial—a large parent study of long‐acting reversible contraception in St Louis, USA.^15^ Those who consented to extending duration of their implants were followed and results of data collected during extension were reported at 2 years of additional use. Investigators collected data on ancillary contraceptive method use as well as presence of participant comorbidities that could impact fertility. In this study, 291 women using the ENG implant elected extended duration: 223 (77%) used the method for more than 12 additional months, and 102 (35%) continued for more than 24 additional months. These women represented a diversity of BMIs with 23% overweight and 52% obese or morbidly obese. In total, study participants contributed 444 women‐years of follow‐up with no documented pregnancies for a calculated 5‐year failure rate of 0 per 100 women‐years.

One study used data from a larger, multicountry RCT of healthy women, at least 6 months postpartum, desiring long‐acting reversible contraception.^20^ Women were initially randomized to the ENG or LNG implant. At the end of 3 years, women in the ENG implant arm were given the choice to continue the ENG implant. Pregnancy rates among women who chose to continue were compared with a nonrandomized control group of women who received a copper‐T intrauterine device (Pregna copper T 380A; Pregna International, Mumbai, India). A subset (n=390) of women randomized to the single‐rod ENG implant consented to extended duration of use; 204 women completed 5 years of use with no reported pregnancies with a calculated Kaplan‐Meier cumulative pregnancy rate of 0.6 per 100 women‐years (95% CI, 0.2–1.8).

All other studies evaluated contraceptive failures cumulatively over the entire use of the ENG implant, without reporting pregnancy rates specifically for extended time periods. A cohort study conducted in China examined women with extended use of the ENG implant. Among the 75 participants who received an ENG implant, 51 completed 4 years of use and there were no reported pregnancies.^21^ Two noncomparative cohort studies evaluated women with 1 year of extended use of the ENG implant. Women who were breastfeeding or recently using another method of contraception were excluded. Sample sizes were small for women completing the fourth year of implant use (n=47 and n=151). No pregnancies were reported in either of these studies.^22^, ^23^

We identified a multicenter pharmacokinetic study investigating extended duration of the LNG implant. Jadelle implants were inserted in 199 women across multiple sites over 7 years. The participants underwent regular blood draws to assess serum LNG levels. Over the course of the study, five women became pregnant: two in the fifth year and three in the seventh year. All five women had serum LNG levels that fell to or below 180 pg/mL and the two women who conceived in the fifth year demonstrated consistently low LNG levels in the three measurements prior to pregnancy.^16^

## 4 DISCUSSION

This systematic review identified six studies published from 1996 to 2017 that met inclusion and exclusion criteria. Five studies evaluated the ENG implant (Implanon) and one evaluated the LNG implant (Jadelle). There were no studies of the Nexplanon or Sino‐Implant II. One study was an RCT, however randomization was to contraceptive method, not duration; the remaining five studies were prospective cohort studies. Three studies separately analyzed contraceptive efficacy among women beyond currently approved duration. All studies were of poor to fair quality by USPTF grading.

These studies enrolled a total of 1075 women and assessed contraceptive efficacy over extended duration of a progestin contraceptive implant with five reported pregnancies, all among women using the LNG implant. Cumulative contraceptive failure rates in these studies are far below the typical use failure rate of many popular user‐dependent methods such as oral contraceptive pills.^1^

This systematic review has several limitations; results should be interpreted with caution. An assessment of risk of bias using the USPSTF guidelines reveals that all six studies are of poor to fair quality. None of the studies compared women with extended duration of use (either randomized or by self‐selection) with women who initiated a new contraceptive implant after completing approved duration. Little demographic information is provided on women who choose to continue using implants beyond the approved duration. The generalizability of these findings is unknown as these populations may differ in baseline fecundity from the general population. Few studies reported use of ancillary contraception such as barrier methods as an exclusion criterion or reported additional analysis to account for increasing participant age and possible decreased fecundity over the study period.

The lack of randomization and small population size within these studies invite concern that participants may not be representative of the population. Inclusion and exclusion criteria for all studies defined participants as medically healthy, limiting generalizability. Additionally, women who elect to extend duration may differ widely from those who replace their implant. Future research should randomize women to extended duration or critically evaluate potential confounders in continuation. Additionally, more pharmacokinetic research is needed on ENG and LNG serum levels as they relate to the outcome of interest: contraceptive failure. Variability in BMI and potential drug interactions should be further evaluated. Moreover, future studies should investigate whether or not extension of duration is a service women desire. While outside the scope of this review, durability of the devices should also be investigated.

If found to be efficacious, the ability to extend duration of contraceptive implants would serve to increase cost‐effectiveness of the device while decreasing barriers for women by reducing the number of visits needed for implant removal and reinsertion. The findings of these studies provide reassuring information for women considering extended duration of ENG implant use; however, more research is needed to confirm findings, particularly among women with medical comorbidities including obesity. At this time, there appears to be insufficient evidence to recommend extended duration of the LNG implant.

When counselling women, providers must take into account the individual patient. Consideration should include a woman's ability to access care, her medical history, and what an unintended pregnancy may mean to the woman. In light of this limited evidence, patients and their providers, in a shared decision‐making model, should weigh risks and benefits of extending implant use beyond the approved duration.

## AUTHOR CONTRIBUTIONS

Both authors were responsible for the design, planning, and execution of the study, data analysis, and manuscript preparation.

## CONFLICTS OF INTEREST

This work was commissioned by the World Health Organization to inform guidance on contraceptive implant duration. The authors have no conflicts of interest.

## Supporting information


**File S1.** PubMed search strategy.

**File S2.** Excluded studies.
