# Supplementary material for: Systematic review of efficacy with extending contraceptive implant duration
Source: Int J Gynaecol Obstet. 2018 Nov 22;144(1):2–8. doi: 10.1002/ijgo.12696 (PMC7379677; doi:10.1002/ijgo.12696)
Supplement: Supplementary file 1 — File S1. PubMed search strategy. [file IJGO-144-2-s001.docx]

File S1

**PubMed search strategy**

"Pregnant Women"[Mesh] OR " Pregnancy"[Mesh] OR " Pregnancy Complications"[Mesh] OR "Maternal Health Services"[Mesh] OR "Mothers"[Mesh] OR "Maternal Death"[Mesh] OR "Maternal-Fetal Relations"[Mesh] OR "Maternal Exposure"[Mesh] OR "Maternal Mortality"[Mesh] OR "Maternal Behavior"[Mesh] OR pregnan* [TIAB] OR Maternal*[TIAB] OR mother*[TIAB] OR child bearing [TIAB] OR perinatal [TIAB] OR Postnatal[TIAB] OR peri-natal [TIAB] OR post natal [TIAB] OR ante natal [TIAB] OR antenatal [TIAB] OR Postpartum [TIAB] OR puerperium [TIAB] OR post partum [TIAB] OR abortion* [TW] OR abortive [TW] OR "Abortion, Induced"[Mesh] AND (Animals [MH] NOT (Humans [MH] AND Animals[MH])) AND "randomized controlled trial"[pt] OR "controlled clinical trial"[pt] OR "clinical trials as topic"[mesh] OR "random allocation"[mesh] OR "double-blind method"[mesh] OR "single-blind method"[mesh] OR "clinical trial"[pt] OR "research design"[mesh:noexp] OR "comparative study"[pt] OR "evaluation studies"[pt] OR "follow-up studies"[mesh] OR "prospective studies"[mesh] OR "cross-over studies"[mesh]OR "clinical trial"[tw] OR ((singl*[tw] OR doubl*[tw] OR trebl*[tw]) AND (mask*[tw] OR blind*[tw])) OR placebo*[tw] OR random*[tw] OR "control"[tw] OR "controls"[tw] OR prospecitv*[tw] OR volunteer*[tw] OR "cohort studies"[mesh] OR "case-control studies"[mesh] OR "comparative study"[pt] OR "risk factors"[mesh] OR "cohort"[tw] OR "compared"[tw] OR "groups"[tw] OR "case control"[tw] OR "multivariable"[tw] AND ("levonorgestrel"[MeSH Terms] OR levonorgestrel[All Fields] OR norplant[All Fields] OR Jadelle[All Fields] OR ("etonogestrel"[MeSH Terms] OR etonogestrel[All Fields] OR Implanon[All Fields] OR Nexplanon[All Fields] OR “sino implant” [TW]).
