# Supplementary material for: Systematic review of efficacy with extending contraceptive implant duration
Source: Int J Gynaecol Obstet. 2018 Nov 22;144(1):2–8. doi: 10.1002/ijgo.12696 (PMC7379677; doi:10.1002/ijgo.12696)
Supplement: Supplementary file 2 — File S2. Excluded studies. [file IJGO-144-2-s002.docx]

File S2

**Excluded studies**

| **Excluded published reference** | **Reason for exclusion** |
| --- | --- |
| Affandi/January 1999 | Did not evaluate contraceptive effectiveness |
| Affandi/March 1999 | Retracted |
| Affandi/January-March 1999 | Did not evaluate contraceptive effectiveness |
| Ali/2015 | Not extended duration |
| Ali/2014 | Abstract |
| Bacon/2005 | Case Series |
| Bahamondes/2014 | Not extended duration |
| Bateman/2004 | Commentary |
| Beerthuizen/2017 | Different outcome of interest |
| Bensouda-Grimaldi/2005 | Not extended duration |
| Cravioto Ma/1997 | Not extended duration |
| Croxatto/2000 | Review |
| Enyindah/2011 | Not extended duration |
| Fischer/2008 | Review |
| French/2000 | Review |
| Hamontri/2007 | Case Report |
| Harrison-Woolrych/2005 | Case Series |
| Jasaitis/2005 | Commentary |
| Kon/1999 | Report |
| Ladipo/2005 | Review |
| Laphikanont/2006 | Not extended duration |
| Maddox/2008 | Review |
| McDonald-Mosley/2010 | Review |
| Meckstroth/2000 | Review |
| Newton/2003 | Review |
| Patni/2009 | Case Report |
| Perry/2015 | Different outcome of interest |
| Rai/2004 | Not extended duration |
| Sivin/1998 | Not extended duration |
| Sivin/1998 | Different outcome of interest |
| Wan/2003 | Subset of larger included study |
| Zheng/1999 | Duplicate publication |
| Total | 32 |
